# Borderline Pathological Celebrity Worship and Impulsive Buying Intent: Mediating and Moderating Roles of Empathy and Gender

**DOI:** 10.3389/fpsyg.2022.823478

**Published:** 2022-05-12

**Authors:** Outong Chen, Xiaojing Zhao, Dongxing Ding, Yifan Zhang, Hongbo Zhou, Ranran Liu

**Affiliations:** ^1^Department of Psychology, Normal College & School of Teacher Education, Qingdao University, Qingdao, China; ^2^Weifang Engineering Vocational College, Qingzhou, China; ^3^Weifang Institute of Technology, Qingzhou, China; ^4^Qingdao Branch, Naval Aeronautical University, Qingdao, China

**Keywords:** borderline pathological celebrity worship, impulsive buying intent, empathy, gender, moderated mediation

## Abstract

The present study constructed a moderated mediation model to examine whether borderline pathological celebrity worship may be associated with higher levels of impulsive buying intent. The mediating role of empathy and the moderating role of gender were also examined. A total of 1,319 participants recruited from a college through the campus network. The results indicated that (1) borderline pathological celebrity worship could positively predict individuals’ impulsive buying intent; (2) the predictive effect of borderline pathological celebrity worship on impulsive buying intent could be mediated by empathy; and (3) gender moderated the predictive effect of borderline pathological celebrity worship on impulsive buying intent. Specifically, relatively higher levels of borderline pathological celebrity worship were found to be associated with higher impulsive buying intent among men. However, this was relatively less significant among women.

**Conclusion:**

The results contribute to previous findings by demonstrating that borderline pathological celebrity worship could affect impulsive buying intent. Moreover, the mediating role of empathy and the moderating role of gender were also revealed.

## Introduction

A celebrity is a person whose name has attention-getting, interest-riveting, and profit-generating value (Rein et al., 1997), and can be from different professions, such as athletes, singers, or writers. However, regardless of celebrities’ professional fields, they maintain their name or status to make money (Turner, 2007). For example, in cooperation with the brand Nike, the hot-selling shoe series “Air Jordan” was launched in 1985; named after Michael Jordan, one of the world’s most famous basketball players. Today, Air Jordan is one of the worlds’ best-selling shoe series. From this perspective, the relationship between celebrities and their fans is complex, as they are not only fans but also potential customers. Some studies have indicated that a celebrity owning or endorsing a specific brand or product may make it more appealing to fans (Atkin and Block, 1983; Knoll and Matthes, 2017; Parayitam et al., 2020; Teng et al., 2020); however, it remains unclear whether customers/fans would purchase those products impulsively. Therefore, this study examined the mechanism underlying the association between borderline pathological celebrity worship and impulsive buying intent, including the mediating effect of empathy and moderating effect of gender.

### Borderline Pathological Celebrity Worship and Impulsive Buying Intent

Previous consumer research has revealed the economic value added by a celebrity. For example, using text mining and sentiment analysis, Kang et al. (2019) found that a celebrity endorser’s attractiveness and expertise can positively impact a firm’s value, while if the endorsement is done by a celebrity who is perceived as untrustworthy, it can negatively impact a firm’s value, from which that firm may find it almost impossible to recover. Similarly, Elberse and Verleun (2012) indicated that signing on to endorse a brand has positive benefits for both the celebrity and the firm. For example, if an athlete endorsing a product makes any major achievement, sales and stock prices of that product may increase significantly. These previous findings indicate that celebrities have an important impact on consumers, especially their fans’ behaviors. The credibility of a celebrity entrepreneur could significantly increase fans’ positive attitudes toward a product, and thus influence their buying intentions (Erdogan, 2010; Teng et al., 2020).

Celebrity worship is defined as a form of involvement in which individuals idolize their favorite celebrities to the point of it resembling “worship” (Brooks, 2021). According to McCutcheon et al. (2002), celebrity worship has both pathological and non-pathological forms. Non-pathological celebrity worship reflects a healthy enthusiasm toward a favorite celebrity; meanwhile, pathological celebrity worship refers to compulsive behaviors and pathological feelings toward a favorite celebrity (Reeves et al., 2012). These pathological symptoms resulted in some negative psychological outcomes. For example, Maltby et al. (2006) revealed that borderline pathological celebrity worship is significantly correlated with clinical personality traits such as fantasy proneness and dissociation. A large-sample study by Sheridan et al. (2007) also suggested that borderline pathological celebrity worship is positively associated with addiction and criminality, as measured by the Eysenck Personality Questionnaire. Notably, borderline pathological celebrity worship is also associated with materialism (Green et al., 2014). Furthermore, Li et al. (2019) primed the participants by asking them to imagine possible positive outcomes after making a purchase and found that materialism could significantly predict online impulsive buying intent. Under this premise, borderline pathological celebrity worship may be related to impulsive buying intent.

Rook (1987) defined impulsive buying as when a consumer experiences a sudden, often powerful, and persistent urge to buy something immediately, typically with diminished regard for the purchases’ consequences. The outcome of impulsive buying usually contradicts long-term goals, such as saving money, leading the buyer to potentially regret having yielded to the impulse (Baumeister, 2002). Vohs and Faber (2007) indicated that an important factor of impulsive buying is a lack of thoughtful consideration of why a person should have a particular product. While studying Bangtan Boys (BTS, a seven-member South Korean boy band) merchandise, Asrie and Misrawati (2020) found a significant correlation between celebrity worship and impulsively buying BTS merchandise among BTS fans. In contrast, the present study aimed to identify a universal relationship between borderline pathological celebrity worship and impulsive buying intent without focusing on a specific celebrity or fans group. Combining the findings of previous studies, it is reasonable to infer that borderline pathological celebrity worship and impulsive buying intent are correlated. Thus, we proposed the following hypothesis:

Hypothesis 1. Borderline pathological celebrity worship will be positively correlated with impulsive buying intent.

### Empathy as a Mediator

Empathy refers to the ability of an individual to understand and feel others’ emotions (Dvash and Shamay-Tsoory, 2014). Through empathy, individuals can connect with the thoughts and feelings of others, which allows them to respond to others in caring and supportive ways. Empathy is highly correlated with celebrity worship. Preliminary research on the relationship between the audience and media characters has shown that empathy is a key factor for the audience to identify with media characters (Cohen, 2001). When the audience identifies with a specific media character, they may be able to empathize with that character, understand them, and share similar feelings, goals, and perspectives. As for celebrity worshipers, there is a specific worship level defined as “a mixture of empathy” (McCutcheon et al., 2002), that is, individuals with borderline pathological celebrity worship tend to over-identify with their favorite celebrity’s career–both successes and failures. More directly, a qualitative study by Cohen (2001) suggested that emotional and cognitive empathy, sharing goals, and absorption are psychological outcomes of celebrity worship. Ang and Chan (2018) suggested that celebrity worship is associated with an individual’s role modeling, empathy, interpersonal relationships, and identity formation. Thus, we may conclude that empathy and borderline pathological celebrity worship are positively correlated.

Moreover, empathy may also be a key factor related to impulsive buying intent. Studying the differences between making decisions for one’s self than for family members, close friends, or strangers, Ziegler and Tunney (2012) found that the more empathetic one’s relationship with another person, the more impulsive decisions that individual might make for them. Impulsive behaviors are generally sudden and unplanned, and interpersonal empathy and emotions may influence such decisions. Thus, the decisions participants made in Ziegler and Tunney’s study were less optimal in the economic sense. Another correlational study revealed that emotional intelligence, which involves empathy, is correlated with impulsive buying behavior (Zia et al., 2018). The more empathic the consumers are, the more impulsive their buying behaviors are. Although the mechanism underlying the relationship between empathy and impulsive buying intent is still uncertain, we can preliminarily infer that there is a correlation between these two variables. Therefore, we proposed the second hypothesis:

Hypothesis 2. Empathy could mediate the predictive effect of borderline pathological celebrity worship on impulsive buying intent.

### Gender as a Moderator

There may be a significant difference in celebrity worship between men and women. McCutcheon et al. (2002) suggested that men and women have different patterns of celebrity worship. For example, both men and women often mention actors as their favorite celebrities; however, men tend to select musicians far less often than women and instead focus on athletes. There are also pieces of evidence that suggest that women are more likely to select a favorite celebrity of the opposite gender than men (Zsila et al., 2021). In conclusion, men and women have different patterns of celebrity worship, both healthy and borderline pathological. Thus, we can infer that there may be a gender difference between the relationship of borderline pathological celebrity worship and impulsive buying intent, but such conclusions require further evidentiary support.

Furthermore, gender may also be a predictive factor of impulsive buying intent. Lin and Lin (2005) reported that women are significantly more likely to show impulsive buying intent than men. This conclusion has been supported by several other studies. For example, in studying the relationship between emotional intelligence and impulsive behavior, Zia et al. (2018) found that women are more likely than men to be impulsive buyers. Lins (2012) posited that the difference between women and men regarding impulsive buying intent may partially reflect gender differences in consumption habits, intentions, and more importantly, decision-making processes. However, previous researchers have also suggested that the idea that women are more likely to make impulsive purchases than men is a negative stereotype (Hwang and Lee, 2015). Thus, based on previous research, we proposed the third hypothesis:

Hypothesis 3. Gender may moderate the relationship between borderline pathological celebrity worship and impulsive buying intent.

### The Present Study

**FIGURE 1:**
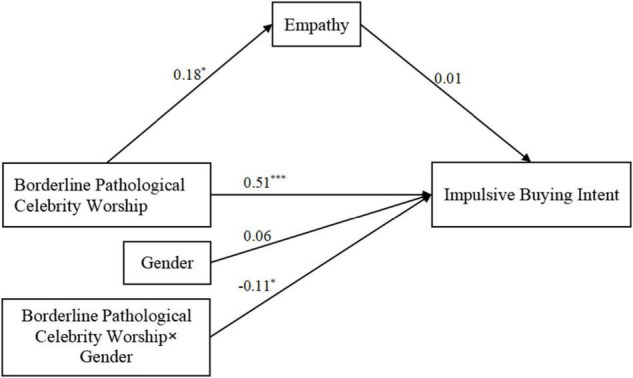
Theoretical moderated mediation model.

The present study aimed to explore the relationship between borderline pathological celebrity worship and impulsive buying intent. Furthermore, we hypothesized that this relationship would be mediated by empathy and moderated by gender. Based on suggestions by Hayes (2013) regarding the combination of mediation and moderation models, if empathy mediates the relationship between borderline pathological celebrity worship and impulsive buying intent, and gender moderates this relationship, a moderated mediation model is thus constructed. The proposed moderated mediation model is presented in Figure 1.

## Materials and Methods

### Participants

A total of 1,319 participants (480 men and 818 women and data were missing for 21 participants; age range: 16–30 years; *M* = 19.21, *SD* = 2.39) recruited from a college through the campus network in October 2021. They completed a series of online questionnaires in random order, including the questionnaires used in the current study. All questionnaires were filled in anonymously to ensure the study’s validity. On average, the participants took approximately 1,514 s to complete the survey. All participants completed the questionnaires sufficiently; therefore, no data were excluded. Missing data were handled as missing data with mean imputation (Little and Rubin, 2002). After each participant completed the questionnaires, they were thanked and given a notebook as a small gift. The current study is approved by the Human Research Ethics Committee for Non-clinical Faculties at School of Psychology, Qingdao University (No. 2021-2-003). participants provided written informed consent prior to the study, and all procedures were performed in accordance with the Declaration of Helsinki.

### Measurements

#### Celebrity Attitude Scale

The Celebrity Attitude Scale (CAS) was originally compiled by McCutcheon et al. (2002) to measure individual celebrity worship behavior. The Borderline Pathological Subscale of a revised version of the CAS (Peng et al., 2010) was adopted in this study to measure participants’ borderline pathological celebrity worship levels. Sample item is “I would gladly die in order to save the life of my favorite celebrity.” Participants were asked to respond to five items using a scale that ranged from 1 (strongly disagree) to 5 (strongly agree), with higher scores representing higher levels of borderline pathological celebrity worship. This scale showed adequate internal consistency in this study (Cronbach’s α = 0.81).

#### Impulsive Buying Intent Scale

The Impulsive Buying Intent Scale (IBIS) was adopted from Lv et al. (2015) to measure participants’ impulsive buying intent in the present study. The IBIS consists of two dimensions: cognitive impulsive buying intent (13 items; e.g., “I will buy something to improve my mood”) and affective impulsive buying intent (13 items; e.g., “I usually decide to buy something without too much thinking”). Participants were asked to respond to each item on a scale ranging from 1 (never) to 5 (all the time). The total score of the IBIS was calculated, with higher scores representing more impulsive buying intent. Items 2, 7, 9, 13, 21, and 25 were reverse scored. In the present study, Cronbach’s α was 0.89 for the IBIS.

#### Interpersonal Reactivity Index

Participants’ empathy in the present study was measured using a revised and localized version of the Interpersonal Reactivity Index (IRI) (Rong et al., 2010). The revised IRI consists of 22 items across four dimensions: fantasy (6 items; e.g., “When I am reading an interesting story or novel, I imagine how I would feel if the events in the story were happening to me”), perspective taking (5 items; e.g., “I sometimes try to understand my friends better by imagining how things look from their perspective”), empathic concern (6 items; e.g., “I often have tender, concerned feelings for people less fortunate than me”), and personal distress (5 items; e.g., “Being in a tense emotional situation scares me”). Participants assessed each item on the IRI using a scale ranging from 1 (never) to 5 (all the time). The total score of the IRI was calculated, with higher scores representing better empathy. Notably, Items 2, 5, 10, 11, and 14 were reverse scored. The IRI showed adequate internal consistency in this study (total scale: Cronbach’s α = 0.82).

### Data Analysis

Data were analyzed using the Statistical Package for Social Sciences (SPSS) version 25. Descriptive statistics from SPSS were used to characterize the participants’ demographics. Pearson’s correlation analysis was used to examine the relationships between borderline pathological celebrity worship, impulsive buying intent, and empathy and the related gender differences. Additionally, we used the SPSS PROCESS macro (Hayes, 2013) to test our hypothesized moderated mediation model. First, the raw scores were transformed to z-scores before testing the moderated mediation effect to obtain the standardized regression coefficients; gender was coded (male = 1; female = 2). Second, PROCESS Model 4 was used to test the mediation effect of empathy. Third, PROCESS Model 59 was used to test the full moderated mediation model. Specifically, the bootstrapping method was applied to test the effects’ significance to obtain robust standard errors for parameter estimation (Chi et al., 2019). This method produced 95% bias-corrected confidence intervals (CIs) for these effects from 1,000 resamples of the data. CIs that do not contain zero indicate significant effects.

## Results

### Common Method Bias

The questionnaires were collected anonymously, and some reversed scores were set to control for possible common method bias. Additionally, Harman’s single factor method was used during data analysis. Exploratory factor analysis obtained 20 factors; the first factor explained 21.32% of the variance, which was less than 40%. This result indicated that no serious common method bias existed in the present study.

### Bivariate Analyses

As shown in Table 1, the bivariate analysis results indicated that borderline pathological celebrity worship was positively correlated with empathy (*r* = 0.22, *p* < 0.001, 95% CI [0.17, 0.27]) and impulsive buying intent (*r* = 0.36, *p* < 0.001, 95% CI [0.32, 0.41]), while empathy was also positively correlated with impulsive buying intent (*r* = 0.16, *p* < 0.001, 95% CI [0.11, 0.22]). Further, an independent sample *t*-test showed that the empathy of male participants (*M* = 91.40, *SD* = 9.93) was significantly lower than that of female participants (*M* = 95.10, *SD* = 11.36), *t* = −5.88, *p* < 0.001, Cohen’s *d* = −0.34, 95% CI [−2.45, −4.89]. The borderline pathological celebrity worship of male participants (*M* = 16.10, *SD* = 6.22) was significantly higher than that of female participants (*M* = 14.70, *SD* = 5.38), *t* = 4.25, *p* < 0.001, Cohen’s *d* = 0.24, 95% CI [0.75, 2.04]. No other significant results were obtained in this analysis.

**TABLE 1:** Descriptive statistics and inter-correlations between variables.

Variables	*M*	*SD*	1	2	3	4
1. Borderline pathological celebrity worship	15.17	5.74	1	0.22***	0.36***	0.08**
2. Empathy	93.64	10.98		1	0.16***	−0.07*
3. Impulsive buying intent	64.32	17.93			1	0.11**
4. Age	19.21	2.37				1

M = mean; SD = standard deviation; *p < 0.05, **p < 0.01, ***p < 0.001.

### Mediation Test for Empathy

**FIGURE 2:**
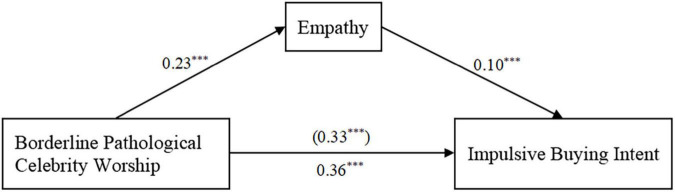
Test of the mediating effect of empathy in the association between borderline pathological celebrity worship and impulsive buying intent. The numbers are standardized regression coefficients. Paths between controlled variable (age) and each of the variables in the model are not displayed, ***p < 0.001.

The mediating effect of empathy was tested using SPSS PROCESS Model 4. After controlling for age, the results showed that borderline pathological celebrity worship had a significant predictive effect on impulsive buying intent (β = 0.36, *t* = 13.88, *p* < 0.001), which remained significant even after entering empathy into the model as a mediating variable (β = 0.33, *t* = 12.74, *p* < 0.001). Further, borderline pathological celebrity worship had a significant predictive effect on empathy (β = 0.23, *t* = 8.46, *p* < 0.001), and the latter had a significant predictive effect on impulsive buying intent (β = 0.10, *t* = 3.67, *p* < 0.001). As hypothesized, empathy partially mediated the significant predictive effect of borderline pathological celebrity worship on impulsive buying intent. The direct effect size was 0.33, 95% CI [0.28, 0.39], the mediation effect was 0.03, 95% CI [0.01, 0.04], accounting for 91.67 and 8.33% of the total effect, respectively (Table 2 and Figure 2).

**TABLE 2:** Testing the mediation effect of empathy.

Predictors (IV)	Model 1 (DV: Impulsive buying intent)		Model 2 (DV: Empathy)		Model 3 (DV: Impulsive buying intent)	
			
	β	*t*	β	*t*	β	*t*
Borderline pathological celebrity worship	0.36	13.88***	0.23	8.46***	0.33	12.74***
Empathy					0.10	3.67***
Age	0.08	3.31***	−0.09	−3.20**	0.09	3.64***
*R* ^2^	0.14		0.06		0.15	
*F*	105.84***		39.12***		75.73***	

Variables in the models are standardized; IV, independent variable; DV, dependent variable; **p < 0.01, ***p < 0.001.

### Moderated Mediation Model Testing

**FIGURE 3:**
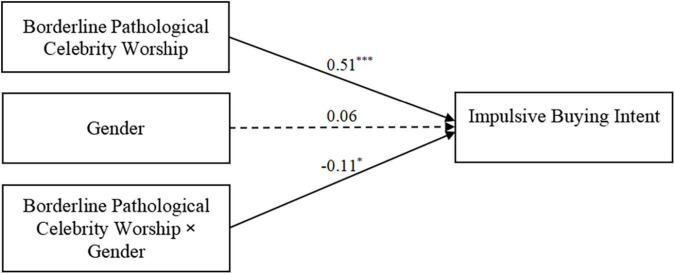
Test of the moderating effect of gender on the direct association between borderline pathological celebrity worship and gender. The numbers are standardized regression coefficients. The dotted line indicates a non-significant relationship. Paths between controlled variables (age) and each of the variables in the models are not displayed, *p < 0.05, ***p < 0.001.

**FIGURE 4:**
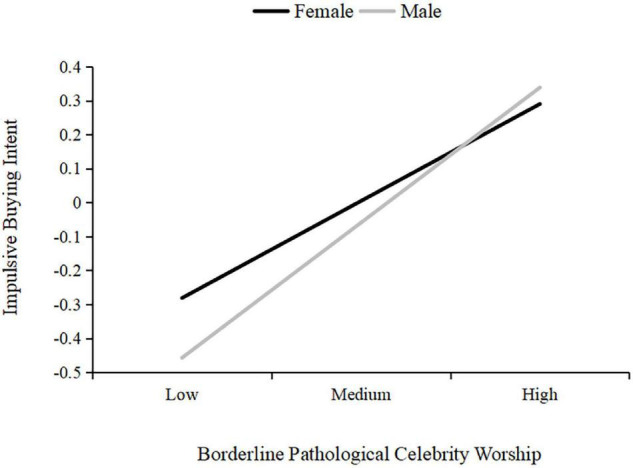
The moderating effect of gender on the relation between borderline pathological celebrity worship and impulsive buying intent. Borderline pathological celebrity worship was graphed for two levels: 1 standard deviation above the mean was high level and 1 standard deviation below the mean was low level. Variables in the models are standardized.

In order to test all the potential moderating effect of gender, the moderated mediation model was tested by adopting SPSS PROCESS Model 59. As shown in Table 3 and Figures 3, 4, the results indicated that borderline pathological celebrity worship had a significant predictive effect on empathy (β = 0.18, *t* = 2.05, *p* = 0.040) and impulsive buying intent (β = 0.51, *t* = 5.77, *p* < 0.001). Empathy (β = 0.01, *t* = 0.07, *p* = 0.946) and gender (β = 0.06, *t* = 1.16, *p* = 0.248) failed to individually predict impulsive buying intent; Moreover, an interaction between borderline pathological celebrity worship and gender was significant (β = −0.11, *t* = −2.09, *p* = 0.037). As shown in Figure 4, simple slope analysis indicated that the conditional direct effect of borderline pathological celebrity worship on impulsive buying intent among both female (*b*_simple_ = 0.29, *p* < 0.001, 95% CI [0.22, 0.36]) and male participants (*b*_simple_ = 0.40, *p* < 0.001, 95% CI [0.32, 0.48]) were both significant. The effect is stronger among males than females. However, the conditional indirect effect of borderline pathological celebrity worship on impulsive buying intent was only significant among female (effect = 0.03, 95% CI [0.01, 0.05]) but not male participants (effect = 0.01, 95% CI [−0.01, 0.04]). No other significant interactions were obtained in this analysis.

**TABLE 3:** Testing the moderated mediation model.

Predictors (IV)	Model 1 (DV: Empathy)		Model 2 (DV: Impulsive Buying Intent)	
		
	β	*t*	β	*t*
Borderline pathological celebrity worship	0.18	2.05*	0.51	5.77***
Empathy			0.01	0.07
Gender			0.06	1.16
Borderline pathological celebrity worship × gender			−0.11	−2.09*
Age	−0.09	−3.53***	0.09	3.40***
*R* ^2^	0.10		0.15	
*F*	33.92***		38.15***	

Variables in the models are standardized; IV, independent variable; DV, dependent variable; *p < 0.05, **p < 0.01, ***p < 0.001.

## Discussion

The present study investigated the relationship between borderline pathological celebrity worship and impulsive buying intent. Additionally, the mediating role of empathy and the moderating role of gender were also investigated. The results revealed that borderline pathological celebrity worship could positively predict impulsive buying behavior. Furthermore, the predictive effect was mediated by empathy and moderated by gender. Specifically, higher levels of borderline pathological celebrity worship led to greater empathy, which, in turn, led to more impulsive buying intent. This effect is relatively stronger among men than women.

The present study demonstrated that borderline pathological celebrity worship is associated with impulsive buying intent. This is consistent with previous findings on the economic relationships between celebrities and their fans. Previous research showed that celebrities could influence higher sales volume, irrespective of whether they owned or simply endorsed a brand or product (Elberse and Verleun, 2012; Teng et al., 2020). Endorsements from celebrities with better reputations could positively affect a product, brand, or firm, and vice versa. It seems that celebrities have an impact on the sale of merchandise, especially when worshiping a celebrity to a borderline pathological level. A study on BTS fans indicated that the higher their level of celebrity worship, the greater their impulsive buying intent of BTS merchandise (Asrie and Misrawati, 2020). However, previous research has focused more on one-to-one correlations of how a specific celebrity affects a specific brand and a specific fan group. The present results contribute to previous findings by focusing on a specific level of celebrity worship, borderline pathological level, and revealing that borderline pathological celebrity worship may provoke impulsive buying intent. This impulsive buying intent could affect daily consumer behavior extensively, rather than leading them to focus on some specific merchandise connected to specific celebrities.

The present study further showed that the predictive effect of borderline pathological celebrity worship on impulsive buying intent is mediated by empathy. Cohen (2001) suggested that when watching a film or show, the audience tends to identify and empathize with a character, thereby understanding and sharing similar feelings, goals, and perspectives with that character. Thus, a higher ability to empathize may be a psychological consequence of celebrity worship (Ang and Chan, 2018). McCutcheon et al. (2002) presented a three-dimensional celebrity worship model that suggests there is a specific worship level, which refers to borderline pathological celebrity worship, defined as “a mixture of empathy”. This refers to the tendency of some fans to regard their worshiped celebrities’ careers (both successes and failures) as their own. They are happy if the celebrity achieves something, such as winning a prize, but also feel upset if the celebrity fails at something. Nevertheless, previous studies generally argue that emotional and cognitive empathy, sharing goals, and absorption are psychological outcomes of celebrity worship (Cohen, 2001).

Regarding the relationship between empathy and impulsive buying intent, Ziegler and Tunney (2012) found that decisions are more impulsive when made for one’s self, family members, or close friends rather than for strangers. Thus, Ziegler and Tunney posited that empathy is involved in the decision-making process, causing decisions to be more impulsive. While studying emotional intelligence, Zia et al. (2018) revealed that empathy, when measured as a dimension of emotional intelligence, is positively correlated with impulsive buying intent. Thus, it could be inferred that when emotions are involved, individuals tend to make less rational, optimal, or planned decisions. Therefore, relatively higher empathy may associated with more impulsive buying intent. These results supported the present study in that the predictive effect of borderline pathological celebrity worship on impulsive buying intent is mediated by empathy.

Moreover, we found that the predictive effect of borderline pathological celebrity worship on impulsive buying intent is moderated by gender. The results of the *t*-test in the current study indicated that women have significantly stronger impulsive buying intent than men, but the results of the subsequent simple slope analysis were more complex. Women demonstrate a higher tendency to be impulsive buyers than men at lower levels of borderline pathological celebrity worship. However, the gender difference in the impulsive buying intent of men and women was no longer significantly different at higher levels of borderline pathological celebrity worship. As borderline pathological celebrity worship levels in men increased, their impulsive buying intent changed from less than to more than that of women. Preliminary findings of the current findings seem to fit the existing stereotype that women are more likely to make impulsive purchases than men (Lin and Lin, 2005; Hwang and Lee, 2015). Compared with men, women are often perceived as having a stronger desire to be admired and be more prone to impulsive purchases of fashion and beauty products (Zia et al., 2018). Previous studies have even discussed in detail women’s consumption habits, intentions, and decision-making processes that differ from those of men (Lins, 2012). The present findings revealed a similar result, that at lower levels of borderline pathological celebrity worship, women are more impulsive buyers that men. However, men with high levels of borderline pathological celebrity worship are as likely as women to make impulsive buying decision. Thus, it is hard to conclude which gender is more likely to engage in impulsive buying behavior. Moreover, the different patterns of borderline pathological celebrity worship on impulsive buying intent among women and men need to be studied further.

Aside from impulsive buying intent, previous studies have also indicated that celebrity worship differs between men and women. Zsila et al. (2020) measured celebrity worship using the CAS and found the levels to be higher among women than men. Furthermore, men and women have been shown to have different patterns of celebrity worship behaviors. For example, some researchers suggested that while men and women often show similarities in relation to celebrity worship of actors, men typically prefer athletes, and women are more likely to prefer musicians (McCutcheon et al., 2002). The current findings, however, revealed no significant gender differences in borderline pathological celebrity worship but did indicate that the relationship between celebrity worship and impulsive buying intent is moderated by gender.

## Limitations

The current study has some limitations. First, the conclusions were based only on questionnaire measures. Thus, stronger evidence from behavioral and cognitive experiments is needed to consolidate the causal inferences. Second, the moderated mediation model was tested only on one population group of young adults. More adolescent samples from other geographic locations and ethnic groups are needed to validate and generalize the present conclusions. Third, the present discussion leaves an open question regarding the mechanism underlying the relationship between empathy and impulsive buying intent. We only attempted to surmise a possibility based on previous findings; however, this needs further clarification based on empirical research.

## Conclusion

This study’s findings indicate that borderline pathological celebrity worship is related to impulsive buying intent, which is mediated by empathy. Furthermore, gender moderates the relationship between borderline pathological celebrity worship and impulsive buying intent. Specifically, relatively higher levels of celebrity worship are associated with more impulsive buying intent, and this relationship is stronger among men than women.

## Data Availability Statement

The original contributions presented in the study are included in the article/supplementary material, further inquiries can be directed to the corresponding author.

## Ethics Statement

The studies involving human participants were reviewed and approved by the Ethics Review form for Studies at the Department of Psychology, Qingdao University. Written informed consent to participate in this study was provided by the participants’ legal guardian/next of kin.

## Author Contributions

OC and XZ designed the research. XZ, DD, YZ, and HZ collected the data. OC wrote the manuscript. RL proofread the manuscript. All authors contributed to the article and approved the submitted version.

## Conflict of Interest

The authors declare that the research was conducted in the absence of any commercial or financial relationships that could be construed as a potential conflict of interest.

## Publisher’s Note

All claims expressed in this article are solely those of the authors and do not necessarily represent those of their affiliated organizations, or those of the publisher, the editors and the reviewers. Any product that may be evaluated in this article, or claim that may be made by its manufacturer, is not guaranteed or endorsed by the publisher.
